# Benchmarking machine learning approaches to predict radiation-induced toxicities in lung cancer patients

**DOI:** 10.1016/j.ctro.2023.100640

**Published:** 2023-05-19

**Authors:** Francisco J. Núñez-Benjumea, Sara González-García, Alberto Moreno-Conde, José C. Riquelme-Santos, José L. López-Guerra

**Affiliations:** a Innovation & Data Analysis Unit, Institute of Biomedicine of Seville, IBiS/Virgen Macarena University Hospital/CSIC/University of Seville, Seville, Spain; b Institute of Biomedicine of Seville, IBIS/Virgen del Rocío University Hospital/CSIC/University of Seville, Seville, Spain; c Department of Computer Languages and Systems, University of Seville, Seville, Spain; d Radiation Oncology Department, Institute of Biomedicine of Seville, IBIS/Virgen del Rocío University Hospital/CSIC/University of Seville, Seville, Spain

**Keywords:** Lung Neoplasms, Radiation-induced toxicity, Machine Learning, Learning Health System, Predictive Models

## Abstract

**Background and purpose:**

Radiation-induced toxicities are common adverse events in lung cancer (LC) patients undergoing radiotherapy (RT). An accurate prediction of these adverse events might facilitate an informed and shared decision-making process between patient and radiation oncologist with a clearer view of life-balance implications in treatment choices. This work provides a benchmark of machine learning (ML) approaches to predict radiation-induced toxicities in LC patients built upon a real-world health dataset based on a generalizable methodology for their implementation and external validation.

**Materials and Methods:**

Ten feature selection (FS) methods were combined with five ML-based classifiers to predict six RT-induced toxicities (acute esophagitis, acute cough, acute dyspnea, acute pneumonitis, chronic dyspnea, and chronic pneumonitis). A real-world health dataset (RWHD) built from 875 consecutive LC patients was used to train and validate the resulting 300 predictive models. Internal and external accuracy was calculated in terms of AUC per clinical endpoint, FS method, and ML-based classifier under analysis.

**Results:**

Best performing predictive models obtained per clinical endpoint achieved comparable performances to methods from state-of-the-art at internal validation (AUC ≥ 0.81 in all cases) and at external validation (AUC ≥ 0.73 in 5 out of 6 cases).

**Conclusion:**

A benchmark of 300 different ML-based approaches has been tested against a RWHD achieving satisfactory results following a generalizable methodology. The outcomes suggest potential relationships between underrecognized clinical factors and the onset of acute esophagitis or chronic dyspnea, thus demonstrating the potential that ML-based approaches have to generate novel data-driven hypotheses in the field.

## Highlights

• Prediction of RT-induced toxicities is important for an informed decision-making process in lung cancer.
• Application of ML approaches to support this task lacks robust validation settings.
• This work provides a generalizable methodology for benchmarking machine learning approaches for this purpose.
• 300 predictive models have been validated following this methodology.
• It shows potential to generate novel data-driven hypotheses in the field.

## Graphical abstract

**Figure:**
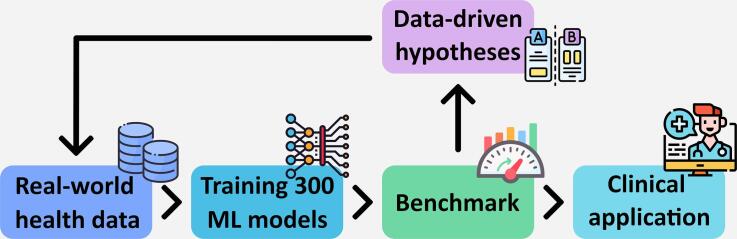

## Introduction

Despite the advances made around improving the accuracy and efficacy of radiotherapy (RT), organs and tissues surrounding the tumor might be affected by the high-energy radiation beam, thus generating an inflammatory response in the affected volumes that may trigger the onset of a number of RT-induced toxicities. The management of the chances for the onset of these toxicities is recognized as one of the most limiting factors during the RT planning [1], preventing an adequate radiation dose to be delivered for a better tumor response, which would translate into potentially improved overall survival for the patient. In addition, RT-induced toxicities may become chronic conditions impairing the patient’s health-related quality of life (HRQoL) in the long-term. Therefore, patients and radiation oncologists are often required to make trade-offs between HRQoL and survivorship in a shared decision-making context, being the baseline HRQoL and future expectations of life the key determinants of preference for the patients [2]. In this context, a personalized and accurate prediction of potential RT-induced toxicities onset would be of great help for patients and oncologists to better understand the life-balance implications of their choices about the treatment options available.

Humans respond to RT in an individually variable manner [3], thus hindering the accuracy of generalized prediction models when applied to specific cases. Besides, published literature reflects a high level of variability in treatment regimes, populations, definition of acute or late toxicities, and accuracy of the reported outcomes, raising concerns about the quality of the evidence generated.

A set of guiding principles for developing ML-based prediction models for RT-induced toxicities was proposed by Kang et al. [4] as follows: (i) consider both dosimetric and non-dosimetric predictors; (ii) manually curate predictors before automated analysis; (iii) select a method for automated predictor selection; (iv) consider how predictor multicollinearity is affecting the model; (v) correctly use cross-validation to improve prediction performance and generalization to external data; (vi) provide model generalizability with external data sets when possible; and (vii) assess multiple models and compare results with established models. Isaksson et al. [5] recently published an overview of ML-based prediction methodologies applied to RT-induced toxicity, in which authors emphasized on the lack of comparability between predictive models due to heterogeneous and not fully validated performance reporting methods, which remains unsolved up to date. Finally, according to the Transparent Reporting of a multivariable prediction model for Individual Prognosis Or Diagnosis (TRIPOD) statement [6], it is highly advisable to assess predictive models performance with an external validation cohort.

The application of ML methods to predict RT-induced toxicity has been challenging the research community for more than a decade. Back in 2007, Dehing-Oberije et al. [7] made a first attempt to apply ML techniques to build a model for the prediction of lung toxicity based on dosimetric, demographics, and clinical variables, achieving an AUC = 0.67 with a multivariate model derived from a cohort of 376 LC patients including WHO-performance scale, FEV1, smoking status, total radiation dose and V20 variables. An interesting finding was that variables representing patient characteristics had a higher predictive power than dosimetric parameters alone. Recently, different ML application approaches have been reported in the literature targeting the prediction of RT-induced toxicities. Lee et al. [8] included genetic information and applied a preconditioned RF algorithm to predict late toxicity in different genitourinary-related endpoints after removing uncorrelated features based on the P values yielded by an univariate analysis for each endpoint, achieving an AUC = 0.70 for the weak stream endpoint. It is of interest the inclusion of genetic information into the model with the aim to explore the role of several SNPs in the late toxicity onset. Another work involving dosimetric and non-dosimetric variables in the prediction of radiation pneumonitis was published by Cui et al. [9]. Their approach demonstrated the potential for combination of traditional machine learning methods and deep learning techniques when dealing with limited datasets for modeling radiotherapy toxicities. With a combination of 24 handcrafted and automatically selected features gathered from a pool of dosimetric and -omics variables, their model achieved an AUC = 0.83. Another study [10] in a modestly sized patient cohort (203 NSCLC patients) used dosimetric and clinical variables to build a predictive model to provide oncologists with cutoff values that facilitate the RT planning. Although they did not reach a remarkable model accuracy (AUC = 0.66), it is noticeable their effort in providing an explainable ML model to end users. Yu et al. [11] made use of a weighted SVM to extract the 3 best performing features of a dataset made up of 185 NSCLC patients with 42 variables each including detailed information on plasma cytokine levels, besides clinical and dosimetric parameters, achieving an AUC = 0.85 in an external validation cohort.

In this context, this work advances the use of real-world health datasets (RWHD) making use of a generalizable methodology to benchmark 300 ML-based models to predict radiation-induced toxicities in LC patients to provide reliable and data-driven real-world evidence-based insights to facilitate the therapeutic decision making process to patients and oncologists.

## Materials and methods

The RWHD was extracted from 875 consecutive LC patients registered in the S31 registry [12], and included information derived from hospital information systems and manually annotated information during routine consultations at the Radiation Oncology department of Virgen del Rocío University Hospital in Seville (Spain) since 2013. This registry addresses a comprehensive set of clinical concepts (see Appendix A) related to LC patients management. Clinical endpoints were acute (esophagitis, cough, dyspnea, and pneumonitis) and chronic (dyspnea and pneumonitis) RT-induced toxicities graded according CTCAE. Toxicities were categorized as chronic in cases where patients had associated symptomatology for more than three months after starting radiation therapy, and were binarized to 0 (grades 0 and 1) or 1 (grades 2 to 5) motivated by the need to perform a clinical intervention. Only variables collected in the consultation before treatment and dosimetry reports were considered as potential predictors, as the predictive model is intended to be used during RT planning sessions. Qualitative variables were binarized using the one-hot-encoding technique, while quantitative variables were assigned a value of 1 in the case that the original value was above the median, and 0 otherwise. Variables not providing information (those variables having the same value across all the cases or totally correlated with other variables) were removed. Besides, cases and variables with more than 20% of the values missing were dropped from the dataset, and the remaining missing values were imputed via a nonparametric technique for mixed-type data [13]. This produced a final dataset of 573 cases and 464 independent variables which was further subset as shown in Table 1 to develop the predictive models.

**Table 1:** Number of cases per clinical endpoint considered. In brackets, the number of cases labeled as positive toxicity.

Clinical endpoint	Acute				Chronic	
	Esophagitis	Cough	Dyspnea	Pneumonitis	Dyspnea	Pneumonitis
**Internal validation cases**	408 (204)	246 (123)	230 (115)	332 (166)	236 (118)	262 (131)
**External validation cases**	46 (16)	45 (11)	46 (12)	46 (18)	31 (1)	31 (7)
**Total cases**	454 (220)	291 (134)	276 (127)	378 (184)	267 (119)	293 (138)

Five ML-based classifiers were implemented: Support Vector Machine (SVM) [14], k-Nearest Neighborhood (kNN) [15], a feedforward Artificial Neural Network (ANN) [16], a Generalized Linear Model (GLM) [17], and a Naïve Bayes (NB) [18] classifier. Whenever available, hyperparameters were automatically optimized following a grid search approach within the recommended ranges provided in the literature. To reduce datasets dimensionality, the following feature selection (FS) methods were implemented: Correlation-based Feature Selection (CFS) [19], Chi-squared (χ^2^) [20], Boruta [21], Minimum Redundancy-Maximum Relevance (mRMR) [22], Relief [23], Information Gain (IG) [20], and Random Forest (RF) [24]. Besides, two ensemble methods were derived from individual and subsetting FS methods, respectively, keeping those variables selected by the majority of these methods. Finally, an expert oncologist also provided subsets of variables to predict the selected toxicities based on the clinical evidence. Altogether, we trained and tested 5 classifiers combined with 10 FS methods to predict 6 different clinical endpoints, summing up 300 different predictive models in total.

For internal validation purposes, a 10-fold cross-validation strategy was followed. A random undersampling technique was applied to generate a balanced dataset for internal validation. For external validation, a temporal approach was followed. The datasets for external validation were generated with the cases registered after May 2018, 31st. These cases were not used during the training or internal validation steps. Performance was measured in terms of the AUC achieved by each predictive model.

## Results

For each clinical endpoint, the best performing predictive models in terms of AUC are shown in Table 2 along with the number and type of clinical variables employed. Appendix B includes the calibration plots for the best performing predictive models. Appendix C provides the average AUC achieved by all the models grouped by FS method and grouped by ML-based classifier, in order to benchmark their performance, while Appendix D includes the AUC achieved by the 300 models analyzed at both cross-validation and external validation stages.

**Table 2:** Best performing models in terms of AUC for each clinical endpoint and clinical variables considered by the models.

**Clinical endpoint**	**Best model**	**AUC (internal validation)**	**AUC (external validation)**	**N Features**	**Clinical variables**
**Acute esophagitis**	mRMR + GLM	0.85	0.81	69	Age, socioeconomic level, HIV, ethnicity, smoking status, primary symptom, anorexia, weight loss, KPS, height, tumor location, histology, EGFR, ALK, TNM, creatinine, hematocrit, familiar cancer history, QoL, concurrent CT, RT dose (lung, esophagus, heart, GTV, CTV).
**Acute cough**	IG + ANN	0.90	0.77	13	Socioeconomic level, QoL, RT dose (lung, esophagus, heart, GTV, CTV)
**Acute dyspnea**	mRMR + GLM	0.81	0.57	32	Socioeconomic level, COPD, oxygen therapy, primary symptom, anorexia, KPS, height, histology, ALK, TNM, Pulmonary function test, familiar cancer history, QoL, RT dose (lung, esophagus, GTV)
**Acute pneumonitis**	χ^2^ + NB	0.81	0.85	24	Socioeconomic level, dyspnea, cough, histology, TNM, QoL, RT dose (CTV, GTV)
**Chronic dyspnea**	mRMR + GLM	0.87	0.97	19	Socioeconomic level, primary symptom, dysphagia, PET, TNM, ALK, familiar cancer history, QoL, GTV
**Chronic pneumonitis**	mRMR + ANN	0.90	0.73	32	Socioeconomic level, primary symptom, dyspnea, pleuritic pain, PET, tumor location, ALK, TNM, Pulmonary function test, familiar cancer history, QoL, RT dose (CTV, GTV, heart)

## Discussion

For acute esophagitis, the best model achieved an AUC = 0.85 in the internal validation and an AUC = 0.81 in the external validation sample. This result seems to outperform state of the art methods [25]. The model was built using the mRMR FS method followed by the GLM classifier considering a total of 69 features that addressed sociodemographic profile, previous conditions, primary symptoms, performance status, tumor characterization (location, histology, TNM), genomic profile (EGFR and ALK expression), family cancer history, QoL, treatment regime, and RT dose (lung, esophagus, heart, GTV, and CTV). The model also highlighted variables related to creatinine and hematocrit levels that, to the best of our knowledge, have not been linked to RT-induced acute esophagitis onset yet.

For acute cough, the best model achieved an AUC = 0.90 in the internal validation and an AUC = 0.77 in the external validation sample. Based on the IG FS method and the ANN classifier, it made use of 13 features describing socioeconomic level, QoL, and RT dose at lungs, esophagus, and heart. To the best of our knowledge, there are no previous works about validations of predictive models for RT-induced acute cough despite its acknowledged importance and contribution to patients’ QoL [26].

Regarding acute dyspnea, the best model in the internal validation achieved an AUC = 0.81 and, in the external validation sample, an AUC = 0.57. Compared to other predictive models available in the literature [27], [28], the developed model seems to outperform most of them at the cross-validation stage. However, its poor performance in the external validation sample must follow a critical review to assess whether it was due to a lack of generalizability of the model or to a different biasing factor, maybe related to the scarce number of samples used for external validation. This model was built using the mRMR FS method followed by the GLM classifier and included 32 features addressing socioeconomic level, baseline conditions, primary symptom, performance status, tumor characterization, ALK expression, outcomes of pulmonary function tests, family cancer history, QoL, and RT dose at lungs and esophagus.

Regarding acute pneumonitis, the best model achieved an AUC = 0.81 in the internal validation and an AUC = 0.85 in the external validation sample. In terms of performance, the model achieved comparable results to state-of-the-art [29]. This model was built upon a chi-squared FS method followed by a NB classifier, accounting with 24 features describing patient’s socioeconomic level, previous conditions (dyspnea, cough), tumor characterization (histology and TNM), QoL, and RT dose (CTV and GTV).

For chronic dyspnea, the best model achieved an AUC = 0.87 in the internal validation and an AUC = 0.97 in the external validation sample, outperforming state-of-the-art methods [30]. This model was built upon an mRMR FS method followed by a GLM classifier trained with 19 features describing patient’s socioeconomic level, primary symptoms, previous conditions (dysphagia), a PET history record, tumor TNM, ALK expression, familial cancer history, QoL, and GTV. To the best of our knowledge, there are no precedents about ALK expression and its relationship with this toxicity in the reviewed literature.

For chronic pneumonitis, the best model achieved an AUC = 0.90 in the internal validation, while its performance in the external validation sample yielded an AUC = 0.73, which seems to be in keeping with state of the art methods [29]. This model was based on the mRMR FS method and the ANN classifier, trained with 32 features addressing the following clinical variables: socioeconomic level, primary symptom, previous dyspnea, pleuritic pain, outcomes of PET and pulmonary function tests, tumor characteristics (location and TNM), ALK expression, family cancer history, QoL, and RT dose at heart, CTV, and GTV.

Selected variables seem to fairly represent current knowledge about potential predictors for each one of the endpoints studied [30], [31], [32], [33]. An interesting finding of this work is that, according to the models produced, there are several common variables related to RT-induced toxicity prediction. Socioeconomic level, QoL, GTV, and TNM were present in all the models, while ALK expression, CTV, primary symptom, and family cancer history were present in 4 out of 6 models.

## Conclusions

This work aimed at advancing the use of RWHD making use of a generalizable methodology to benchmark 300 ML-based models to predict radiation-induced toxicities in LC patients. The proposed methodology achieved, in most cases, comparable performances to state-of-the art approaches, while demonstrates its potential for scaling up to further FS methods, ML-based classifiers, and RWHD. Besides, this work was able to point out potentially novel clinical predictors for some toxicities. This is of great importance as it demonstrates the potential of ML-based approaches to generate new data-driven hypotheses in the field.

This work has several limitations. The RWHD lacks information from other domains (lifestyle, psychological distress, stigma, health disparities, cognitive impairment, PROMs, radiomics, and patient-generated data from wearable devices) which may have the potential to contribute to the prediction of RT-induced toxicities. The proposed methodology in this work would facilitate the exploitation of this information. Future works should explore the addition of such variables to the RWHD to test their contribution to the overall performance. Despite having implemented an external validation process to generalize the results as much as possible, a potential population bias should be considered given that all LC patients were recruited from a single hospital in southern Spain and, therefore, they are not representative of the different ethnic groups that could be found in other locations. Another limitation related to the localization issue relates to the therapeutic options available in this hospital, which were delivered according to the NCCN guidelines for LC patients. Therefore, conclusions should not be generalized to other clinical settings providing care to different populations and/or following different clinical guidelines.

The models tested included a limited set of ten FS methods and five ML-based classifiers, and hyperparameters tuning was limited to a basic grid search approach. This might have produced sub-optimal outcomes in terms of accuracy, as none of the 300 predictive models tested were manually optimized. This decision was made for the sake of the generalizability of the proposed approach. Future works may involve additional FS methods and other families of ML-based classifiers. In addition, a high variability between internal and external validation performance has been observed. In order to provide a more realistic estimate of models’ accuracy, future works could address the allocation of a larger sample for the external validation dataset.

The use of validated predictive models providing reliable predictions about expected quality of life enables an informed and shared decision making between the patient and the oncologist, facilitating a more personalized treatment choice.

## Patient Consent Statement

All subjects represented in the S31 registry have given their informed consent to make use of their data for the development of this study. This study was approved by the ethics committee of Virgen Macarena & Virgen del Rocio University Hospitals under protocol ID 1641-N-16 ensuring that this research complies to the Helsinki declaration principles, good clinical practices for biomedical research, and regulations on personal data privacy.

## Declaration of Competing Interest

The authors declare that they have no known competing financial interests or personal relationships that could have appeared to influence the work reported in this paper.
