# Supplementary material for: Benchmarking machine learning approaches to predict radiation-induced toxicities in lung cancer patients
Source: Clin Transl Radiat Oncol. 2023 May 19;41:100640. doi: 10.1016/j.ctro.2023.100640 (PMC10213176; doi:10.1016/j.ctro.2023.100640)
Supplement: Supplementary data 1 [file mmc1.docx]

# Appendix A. Clinical concepts included in the S31 registry

- Demographics and socio-economic status.
- Consultation data: history record of consultations including date, specialty, reason for consulting, healthcare professional consulted, and referral center.
- Family history: kinship and cancer localization.
- Personal history: allergies, toxic habits (smoking, alcohol intake), respiratory history (COPD, oxygen therapy), neoplasm history, general history, surgical history, quality of life.
- Medication: current medication, specifying name, dose, units, via, frequency, start and end dates.
- Diagnostic: clinical diagnostic, radiological diagnostic, pathological anatomy, serum tumor markers, genetic tests, tumor staging.
- Physical examination: Karnofsky index, pulmonary and cardiac auscultation findings.
- Treatment: chemotherapy, surgery, radiotherapy and dosimetry.
- Evolution: toxicity and quality of life.
- Response to treatment: date, type of response, clinical test and decision taken.
- Last contact: date, patient status and findings.

Furthermore, this registry aims at reflecting the LC patient journey and, therefore, the information for each patient is collected according to the four main stages of this process in the following documents:

- Consultation report: this document addresses clinical data of the patient before starting the treatment. It includes demographics, consultation data, family history, personal history, medication, diagnostic, physical examination and proposed treatment, among others.
- Dosimetry: This document collects data related to the dosimetry reports from the RT information systems.
- Evolution and clinical course: This document collects information associated with the patient’s evolution right after the treatment, including toxicity, adverse events and relapses.
- Quality of life: This final document incorporates the completed validated psychometrics about health-related quality of life at different points of the patient journey.

**Appendix B. Calibration plots of the best performing predictive models**


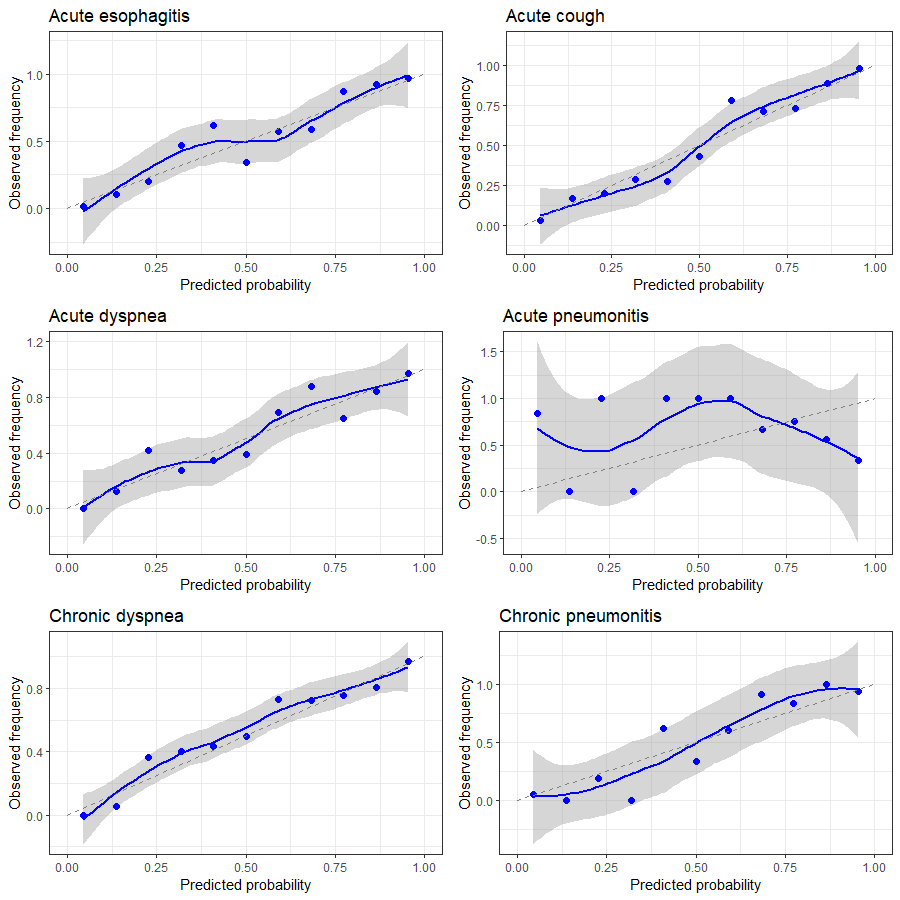


**Figure B.1.** Calibration plots of the best performing predictive models for each clinical endpoint analyzed.

According to the calibration plots, 5 out of the 6 models developed fairly represent a quasi-linear relationship between the predicted probabilities and the observed frequencies found in positive cases. Regarding the acute pneumonitis model, the calibration plot illustrates a well-known issue about the inability of Naive Bayes classifiers to produce calibrated probability estimates^[[1]](#footnote-1)^. This drawback can be overcome making use of calibration models^[[2]](#footnote-2)^.

**Appendix C. AUC per FS method and per ML-based classifier**

## C.1. AUC per FS method

The AUC achieved by each one of the FS methods analyzed disregarding the ML-based classifier and the target endpoint is provided to benchmark their performance. The statistical significance of the differences found among the AUC achieved by the clinical FS method when compared to the other methods has also been calculated for both scenarios (cross-validation and external validation).

**Table C.1**. AUC average and standard deviation during cross-validation and external validation for each one of the FS methods analyzed disregarding the clinical endpoint and the ML-based classifier.

| **FS Method** | **Cross-validation AUC** | **External validation AUC** |
| --- | --- | --- |
| **CFS** | 0.71 (0.11) *** | 0.66 (0.13) |
| **𝜒^2^** | 0.73 (0.10) *** | 0.69 (0.10) ** |
| **Boruta** | 0.74 (0.11) *** | 0.69 (0.10) * |
| **Poll (subsetting)** | 0.73 (0.10) *** | 0.70 (0.12) ** |
| **mRMR** | 0.74 (0.11) *** | 0.67 (0.15) |
| **Relief** | 0.65 (0.05) *** | 0.66 (0.14) |
| **RF** | 0.74 (0.09) *** | 0.66 (0.13) |
| **IG** | 0.72 (0.10) *** | 0.67 (0.11) |
| **Poll (ranking)** | 0.74 (0.10) *** | 0.64 (0.13) |
| **Clinical** | 0.59 (0.04) | 0.62 (0.10) |

* p-value < 0.05 (95% CI).

** p-value < 0.01 (95% CI).

*** p-value < 0.001 (95% CI).

As shown in Table C.1, the performance of the 10 selected FS methods in terms of AUC at both cross-validation and external validation stages has been analyzed, disregarding the clinical endpoints and the ML classifiers employed. The Clinical FS method was considered as the gold standard, as the features were selected by an expert RT oncologist according to the current evidence. These features were, for the esophagitis endpoint: RT dose (mean esophagus dose, V35, V50, and V70), previous digestive conditions, age, KPS, smoking status, TNM, and treatment plan; and for the rest of endpoints: RT dose (mean lung dose and V20), age, previous COPD, previous oxygen therapy, smoking status, KPS, TNM, and treatment plan.


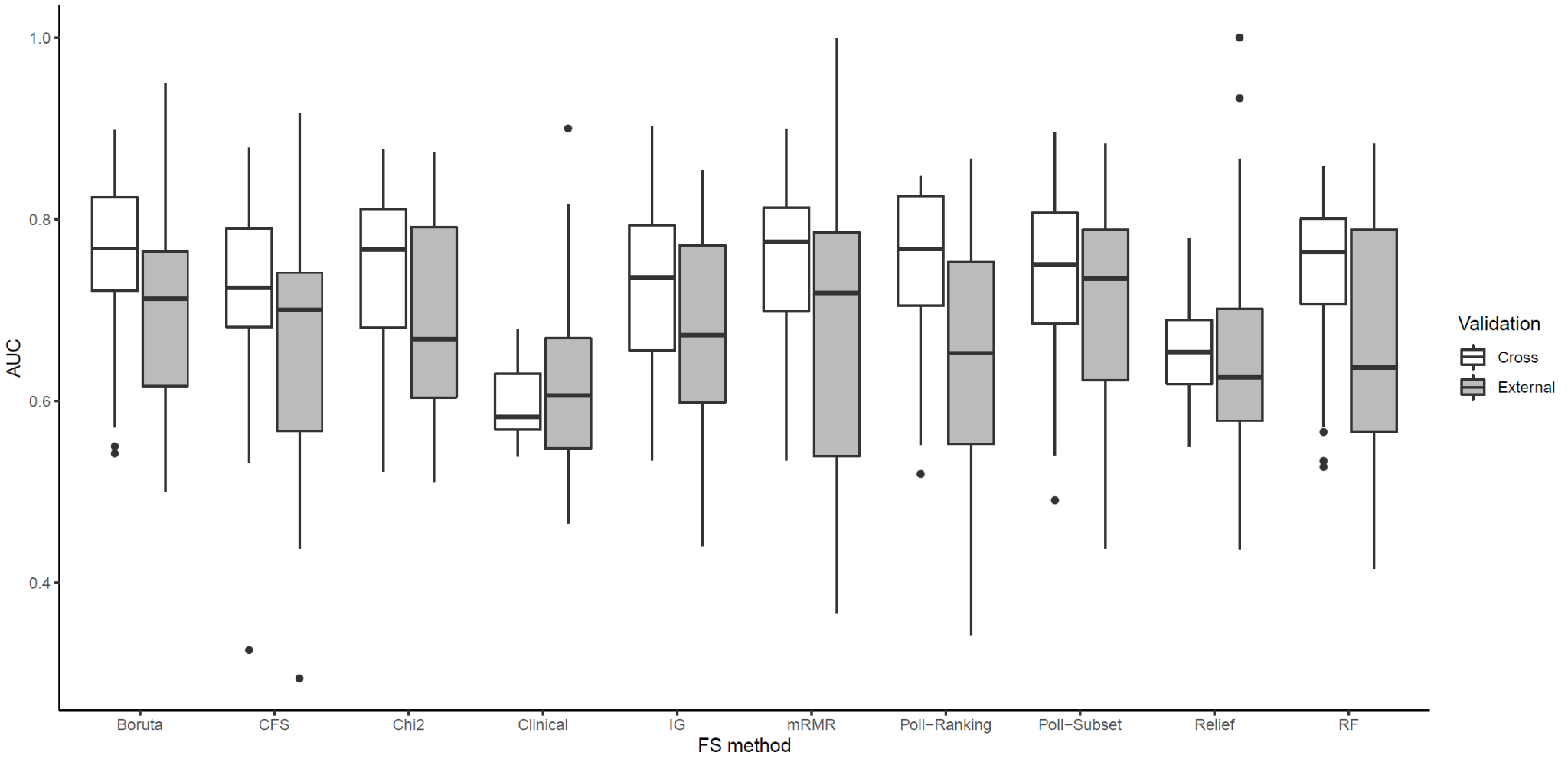


**Figure C.1.** Boxplot representing the AUC distribution for each one of the FS methods included in the analysis after cross-validation and external validation, disregarding the clinical endpoint addressed and the ML-based classifier implemented.

The other nine FS methods analyzed were compared to the Clinical one in order to find out potential statistically significant differences among them. At cross-validation stage, the Clinical FS method achieved an average AUC=0.59 (0.04), while the other 9 FS methods ranged from an average AUC=0.65 (0.05) achieved by the Relief FS method to an average AUC=0.74 (0.09) achieved by the RF FS method. This average AUC was also achieved by the polling-ranking, Boruta, and mRMR FS methods, with a slightly higher standard deviation (0.10, 0.11, and 0.11, respectively). It is remarkable that, at this stage, all the FS methods analyzed achieved statistically significant improvements in their performance when compared to the Clinical FS method (*p*<0.001). At the external validation stage, the Clinical FS method achieved an average AUC=0.62 (0.10), while the other FS methods ranged from an average AUC=0.64 (0.13) achieved by the polling-ranking FS method to an average AUC=0.70 (0.12) achieved by the polling-subsetting FS method. In this case, only three FS methods (𝜒^2^, Boruta, and polling-subsetting) achieved a statistically significant improvement in their performance when compared to the gold standard. In general, the average performance achieved for all the FS methods at the external validation stage was considerably lower than the ones achieved during the cross-validation, which is in line with current evidence^[[3]](#footnote-3)^. It should be noted the high internal variability in the performances achieved by the FS methods, in some cases like mRMR in the external validation cohort ranging from AUC=0.37 to AUC=1.0. This could be motivated because of the involvement of 30 different use cases (the combination 5 ML-based classifiers and 6 clinical endpoints) in this assessment, but it can also provide an idea of the stability of the FS method when compared to the others.

## C.2. AUC per ML-based classifier

In this section, the AUC achieved by each one of the ML-based classifiers analyzed disregarding the FS method and the target endpoint is provided to benchmark their performance.

**Table C.2**. AUC average and standard deviation during cross-validation and external validation for each one of the ML-based classifiers analyzed disregarding the clinical endpoint and the FS method.

| **ML Method** | **Cross-validation AUC** | **External validation AUC** |
| --- | --- | --- |
| **kNN** | 0.57 (0.03) | 0.58 (0.13) |
| **GLM** | 0.77 (0.08) | 0.72 (0.10) |
| **NB** | 0.74 (0.09) | 0.67 (0.14) |
| **SVM** | 0.71 (0.07) | 0.68 (0.10) |
| **ANN** | 0.76 (0.08) | 0.68 (0.10) |

**
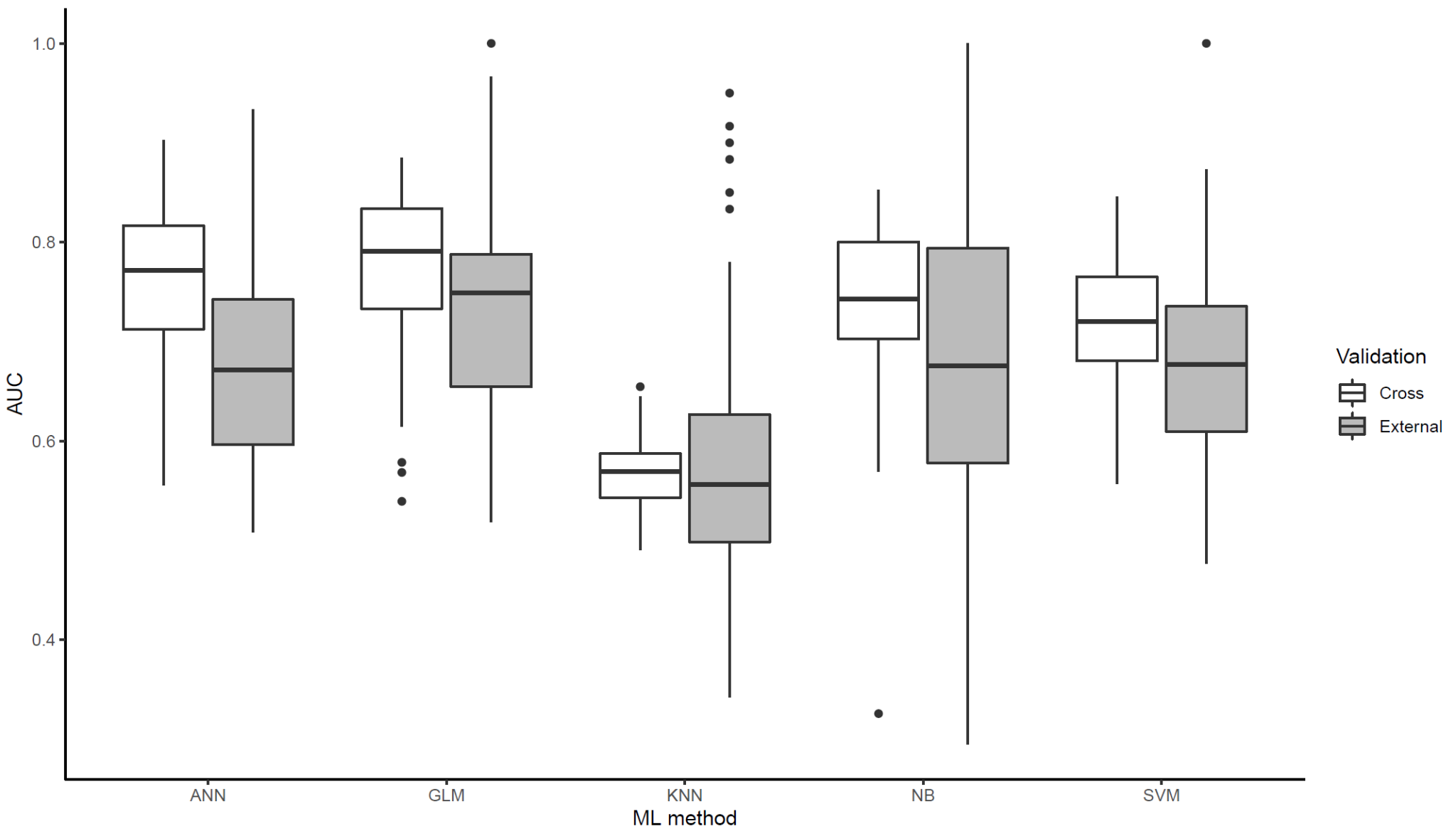
**

**Figure C.2.** Boxplot representing the AUC distribution for each one of the classifiers included in the analysis, disregarding the clinical endpoint addressed and the FS method implemented.

As reported in Table C.2, the performance in terms of average AUC and standard deviation of the ML-based classifiers disregarding clinical endpoints and FS method implemented has been analyzed. At the cross-validation stage, the performance ranged from an average AUC=0.57 (0.03) achieved by the kNN classifier to an average AUC=0.77 (0.08) achieved by the GLM classifier. It should be noted that the other three classifiers (NB, SVM, and ANN) scored an average performance over 0.70. At the external validation stage, the performance ranged from an average AUC=0.58 (0.13) achieved by the kNN classifier to an average AUC=0.72 (0.10) achieved by the GLM classifier. NB, SVM, and ANN classifiers scored a similar average performance (0.67, 0.68, and 0.68, respectively).

These outcomes consistently report about the poor performance of the kNN classifier to predict RT-induced toxicities in general when using a RWHD, which might be related to a high level of noise in the training dataset^[[4]](#footnote-4)^ given its real-world nature. GLM classifier achieved the highest average AUC at both stages (cross-validation and external validation), but closely followed by the NB, SVM, and ANN classifiers at external validation stage, which might imply that the four classifiers seem to adapt better than the kNN to a real-world environment. Stability of the ML-based classifiers benchmarked can be assessed by the internal variability in the performances achieved with the external validation cohort. In this sense, NB showed a very high variability (from AUC=0.29 to AUC=1.00) which may discourage its use in a real-world setting.

**Appendix D. AUC achieved by the 300 predictive models in internal and external validation stages. The number of features used by each combination is shown in brackets.**

Table D.1: AUC and number of features used for each one of the tested combinations of FS method and ML-based classifier during the 10-fold cross-validation for acute esophagitis prediction.

|  | **kNN** | **GLM** | **NB** | **SVM** | **ANN** |
| --- | --- | --- | --- | --- | --- |
| **CFS** | 0.55 (6) | 0.72 (6) | 0.70 (6) | 0.73 (6) | 0.75 (6) |
| **Chi-squared** | 0.55 (43) | 0.82 (43) | 0.72 (43) | 0.68 (43) | 0.78 (43) |
| **Boruta** | 0.58 (30) | 0.84 (30) | 0.77 (30) | 0.72 (30) | 0.83 (30) |
| **Poll (subsetting)** | 0.54 (22) | 0.77 (22) | 0.73 (22) | 0.69 (22) | 0.80 (22) |
| **mRMR** | 0.55 (22) | **0.85 (69)** | 0.72 (9) | 0.71 (78) | 0.76 (65) |
| **Relief** | 0.55 (63) | 0.66 (153) | 0.65 (81) | 0.67 (100) | 0.62 (449) |
| **RF** | 0.57 (116) | 0.79 (81) | 0.73 (11) | 0.73 (22) | 0.80 (18) |
| **IG** | 0.53 (4) | 0.80 (33) | 0.72 (29) | 0.62 (22) | 0.78 (28) |
| **Poll (ranking)** | 0.55 (20) | 0.83 (68) | 0.74 (14) | 0.74 (78) | 0.70 (82) |
| **Clinical** | 0.54 (33) | 0.68 (33) | 0.61 (33) | 0.60 (33) | 0.67 (33) |

Table D.2: AUC and number of features used for each one of the tested combinations of FS method and ML-based classifier during external validation for acute esophagitis prediction.

|  | **kNN** | **GLM** | **NB** | **SVM** | **ANN** |
| --- | --- | --- | --- | --- | --- |
| **CFS** | 0.78 (6) | 0.66 (6) | 0.76 (6) | 0.67 (6) | 0.70 (6) |
| **Chi-squared** | 0.65 (43) | 0.80 (43) | 0.79 (43) | **0.87 (43)** | 0.83 (43) |
| **Boruta** | 0.62 (30) | 0.79 (30) | 0.76 (30) | 0.72 (30) | 0.73 (30) |
| **Poll (subsetting)** | 0.61 (22) | 0.79 (22) | 0.79 (22) | 0.73 (22) | 0.83 (22) |
| **mRMR** | 0.47 (22) | 0.81 (69) | 0.72 (9) | 0.67 (78) | 0.84 (65) |
| **Relief** | 0.44 (63) | 0.81 (153) | 0.69 (81) | 0.68 (100) | 0.60 (449) |
| **RF** | 0.42 (116) | 0.73 (81) | 0.81 (11) | 0.78 (22) | 0.81 (18) |
| **IG** | 0.61 (4) | 0.77 (33) | 0.80 (29) | 0.68 (22) | 0.85 (28) |
| **Poll (ranking)** | 0.53 (20) | 0.85 (68) | 0.82 (14) | 0.77 (78) | 0.77 (82) |
| **Clinical** | 0.47 (33) | 0.75 (33) | 0.67 (33) | 0.63 (33) | 0.82 (33) |

Table D.3: AUC and number of features used for each one of the tested combinations of FS method and ML-based classifier during the 10-fold cross-validation for acute cough prediction.

|  | **kNN** | **GLM** | **NB** | **SVM** | **ANN** |
| --- | --- | --- | --- | --- | --- |
| **CFS** | 0.60 (10) | 0.87 (10) | 0.84 (10) | 0.79 (10) | 0.88 (10) |
| **Chi-squared** | 0.57 (34) | 0.88 (34) | 0.84 (34) | 0.76 (34) | 0.83 (34) |
| **Boruta** | 0.58 (15) | 0.88 (15) | 0.85 (15) | 0.75 (15) | 0.90 (15) |
| **Poll (subsetting)** | 0.61 (13) | 0.87 (13) | 0.84 (13) | 0.74 (13) | **0.90 (13)** |
| **mRMR** | 0.62 (19) | 0.88 (21) | 0.81 (65) | 0.81 (43) | 0.88 (20) |
| **Relief** | 0.64 (53) | 0.72 (34) | 0.71 (36) | 0.78 (131) | 0.71 (41) |
| **RF** | 0.61 (18) | 0.86 (13) | 0.82 (220) | 0.80 (123) | 0.83 (95) |
| **IG** | 0.59 (13) | 0.89 (14) | 0.83 (19) | 0.66 (16) | **0.90 (13)** |
| **Poll (ranking)** | 0.58 (14) | 0.85 (12) | 0.84 (66) | 0.83 (64) | 0.84 (22) |
| **Clinical** | 0.57 (33) | 0.64 (33) | 0.60 (33) | 0.60 (33) | 0.64 (33) |

Table D.4: AUC and number of features used for each one of the tested combinations of FS method and ML-based classifier during external validation for acute cough prediction.

|  | **kNN** | **GLM** | **NB** | **SVM** | **ANN** |
| --- | --- | --- | --- | --- | --- |
| **CFS** | 0.57 (10) | 0.74 (10) | 0.76 (10) | 0.70 (10) | 0.79 (10) |
| **Chi-squared** | 0.51 (34) | 0.78 (34) | 0.81 (34) | 0.58 (34) | 0.66 (34) |
| **Boruta** | 0.61 (15) | 0.77 (15) | 0.82 (15) | 0.72 (15) | 0.75 (15) |
| **Poll (subsetting)** | 0.53 (13) | 0.79 (13) | **0.82 (13)** | 0.67 (13) | 0.74 (13) |
| **mRMR** | 0.37 (19) | 0.75 (21) | 0.46 (65) | 0.53 (43) | 0.66 (20) |
| **Relief** | 0.53 (53) | 0.58 (34) | 0.68 (36) | 0.70 (131) | 0.51 (41) |
| **RF** | 0.57 (18) | 0.79 (13) | 0.61 (220) | 0.61 (123) | 0.52 (95) |
| **IG** | 0.61 (13) | 0.80 (14) | 0.82 (19) | 0.61 (16) | 0.77 (13) |
| **Poll (ranking)** | 0.52 (14) | 0.76 (12) | 0.60 (66) | 0.64 (64) | 0.55 (22) |
| **Clinical** | 0.54 (33) | 0.52 (33) | 0.68 (33) | 0.60 (33) | 0.58 (33) |

Table D.5: AUC and number of features used for each one of the tested combinations of FS method and ML-based classifier during the 10-fold cross-validation for acute dyspnea prediction.

|  | **kNN** | **GLM** | **NB** | **SVM** | **ANN** |
| --- | --- | --- | --- | --- | --- |
| **CFS** | 0.57 (9) | 0.75 (9) | 0.72 (9) | 0.68 (9) | 0.75 (9) |
| **Chi-squared** | 0.61 (23) | 0.78 (23) | 0.77 (23) | 0.72 (23) | 0.77 (23) |
| **Boruta** | 0.57 (15) | 0.74 (15) | 0.73 (15) | 0.72 (15) | 0.73 (15) |
| **Poll (subsetting)** | 0.57 (9) | 0.75 (9) | 0.72 (9) | 0.68 (9) | 0.75 (9) |
| **mRMR** | 0.54 (188) | **0.81 (32)** | 0.74 (48) | 0.77 (57) | 0.79 (53) |
| **Relief** | 0.59 (62) | 0.68 (27) | 0.67 (23) | 0.71 (154) | 0.64 (72) |
| **RF** | 0.62 (234) | 0.76 (42) | 0.70 (31) | 0.80 (256) | 0.76 (173) |
| **IG** | 0.64 (4) | 0.72 (4) | 0.63 (2) | 0.71 (2) | 0.76 (4) |
| **Poll (ranking)** | 0.52 (105) | 0.75 (12) | 0.70 (11) | 0.78 (112) | 0.78 (49) |
| **Clinical** | 0.58 (33) | 0.64 (33) | 0.64 (33) | 0.68 (33) | 0.67 (33) |

Table D.6: AUC and number of features used for each one of the tested combinations of FS method and ML-based classifier during external validation for acute dyspnea prediction.

|  | **kNN** | **GLM** | **NB** | **SVM** | **ANN** |
| --- | --- | --- | --- | --- | --- |
| **CFS** | 0.44 (9) | 0.56 (9) | 0.68 (9) | 0.48 (9) | 0.56 (9) |
| **Chi-squared** | 0.59 (23) | 0.62 (23) | 0.58 (23) | 0.69 (23) | 0.62 (23) |
| **Boruta** | 0.53 (15) | 0.53 (15) | 0.56 (15) | 0.50 (15) | 0.57 (15) |
| **Poll (subsetting)** | 0.44 (9) | 0.56 (9) | 0.68 (9) | 0.48 (9) | 0.56 (9) |
| **mRMR** | 0.49 (188) | 0.57 (32) | 0.52 (48) | 0.64 (57) | 0.57 (53) |
| **Relief** | 0.65 (62) | 0.70 (27) | **0.76 (23)** | 0.73 (154) | 0.60 (72) |
| **RF** | 0.58 (234) | 0.69 (42) | 0.67 (31) | 0.66 (256) | 0.51 (173) |
| **IG** | 0.63 (4) | 0.55 (4) | 0.60 (2) | 0.60 (2) | 0.54 (4) |
| **Poll (ranking)** | 0.63 (105) | 0.68 (12) | 0.75 (11) | 0.61 (112) | 0.60 (49) |
| **Clinical** | 0.47 (33) | 0.61 (33) | 0.57 (33) | 0.56 (33) | 0.65 (33) |

Table D.7: AUC and number of features used for each one of the tested combinations of FS method and ML-based classifier during the 10-fold cross-validation for acute pneumonitis prediction.

|  | **kNN** | **GLM** | **NB** | **SVM** | **ANN** |
| --- | --- | --- | --- | --- | --- |
| **CFS** | 0.53 (4) | 0.71 (4) | 0.33 (4) | 0.68 (4) | 0.69 (4) |
| **Chi-squared** | 0.56 (24) | 0.77 (24) | **0.81 (24)** | 0.69 (24) | 0.76 (24) |
| **Boruta** | 0.54 (14) | 0.80 (14) | 0.80 (14) | 0.60 (14) | 0.77 (14) |
| **Poll (subsetting)** | 0.54 (10) | 0.78 (10) | 0.78 (10) | 0.68 (10) | 0.77 (10) |
| **mRMR** | 0.57 (6) | 0.78 (43) | 0.79 (46) | 0.73 (49) | 0.80 (24) |
| **Relief** | 0.56 (122) | 0.64 (100) | 0.66 (158) | 0.66 (337) | 0.64 (379) |
| **RF** | 0.53 (187) | 0.76 (61) | 0.78 (208) | 0.69 (61) | 0.73 (178) |
| **IG** | 0.54 (7) | 0.79 (13) | 0.77 (12) | 0.68 (9) | 0.79 (8) |
| **Poll (ranking)** | 0.55 (64) | 0.79 (31) | 0.80 (104) | 0.74 (89) | 0.71 (160) |
| **Clinical** | 0.54 (33) | 0.58 (33) | 0.59 (33) | 0.56 (33) | 0.57 (33) |

Table D.8: AUC and number of features used for each one of the tested combinations of FS method and ML-based classifier during external validation for acute pneumonitis prediction.

|  | **kNN** | **GLM** | **NB** | **SVM** | **ANN** |
| --- | --- | --- | --- | --- | --- |
| **CFS** | 0.54 (4) | 0.77 (4) | 0.29 (4) | 0.80 (4) | 0.71 (4) |
| **Chi-squared** | 0.56 (24) | 0.83 (24) | **0.85 (24)** | 0.66 (24) | 0.71 (24) |
| **Boruta** | 0.60 (14) | 0.75 (14) | 0.69 (14) | 0.76 (14) | 0.68 (14) |
| **Poll (subsetting)** | 0.67 (10) | 0.84 (10) | 0.83 (10) | 0.76 (10) | 0.83 (10) |
| **mRMR** | 0.48 (6) | 0.80 (43) | 0.82 (46) | 0.74 (49) | 0.73 (24) |
| **Relief** | 0.59 (122) | 0.64 (100) | 0.59 (158) | 0.62 (337) | 0.66 (379) |
| **RF** | 0.54 (187) | 0.81 (61) | 0.49 (208) | 0.79 (61) | 0.68 (178) |
| **IG** | 0.56 (7) | 0.80 (13) | 0.83 (12) | 0.72 (9) | 0.70 (8) |
| **Poll (ranking)** | 0.55 (64) | 0.74 (31) | 0.69 (104) | 0.56 (89) | 0.59 (160) |
| **Clinical** | 0.47 (33) | 0.59 (33) | 0.54 (33) | 0.54 (33) | 0.61 (33) |

Table D.9: AUC and number of features used for each one of the tested combinations of FS method and ML-based classifier during the 10-fold cross-validation for chronic dyspnea prediction.

|  | **kNN** | **GLM** | **NB** | **SVM** | **ANN** |
| --- | --- | --- | --- | --- | --- |
| **CFS** | 0.63 (7) | 0.81 (7) | 0.79 (7) | 0.77 (7) | 0.81 (7) |
| **Chi-squared** | 0.59 (29) | 0.83 (29) | 0.82 (29) | 0.66 (29) | 0.68 (29) |
| **Boruta** | 0.60 (19) | 0.86 (19) | 0.85 (19) | 0.79 (19) | 0.83 (19) |
| **Poll (subsetting)** | 0.59 (12) | 0.83 (12) | 0.81 (12) | 0.74 (12) | 0.82 (12) |
| **mRMR** | 0.54 (15) | **0.87 (19)** | 0.65 (435) | 0.79 (9) | 0.81 (9) |
| **Relief** | 0.63 (14) | 0.62 (97) | 0.69 (113) | 0.73 (176) | 0.67 (248) |
| **RF** | 0.57 (205) | 0.83 (16) | 0.78 (206) | 0.76 (13) | 0.80 (16) |
| **IG** | 0.65 (9) | 0.80 (10) | 0.79(10) | 0.71 (13) | 0.76 (10) |
| **Poll (ranking)** | 0.58 (21) | 0.83 (13) | 0.78 (252) | 0.76 (15) | 0.82 (17) |
| **Clinical** | 0.54 (33) | 0.54 (33) | 0.59 (33) | 0.57 (33) | 0.56 (33) |

Table D.10: AUC and number of features used for each one of the tested combinations of FS method and ML-based classifier during external validation for chronic dyspnea prediction.

|  | **kNN** | **GLM** | **NB** | **SVM** | **ANN** |
| --- | --- | --- | --- | --- | --- |
| **CFS** | 0.92 (7) | 0.73 (7) | 0.57 (7) | 0.70 (7) | 0.73 (7) |
| **Chi-squared** | 0.83 (29) | 0.57 (29) | 0.63 (29) | 0.63 (29) | 0.60 (29) |
| **Boruta** | 0.95 (19) | 0.77 (19) | 0.67 (19) | 0.77 (19) | 0.77 (19) |
| **Poll (subsetting)** | 0.88 (12) | 0.67 (12) | 0.60 (12) | 0.80 (12) | 0.67 (12) |
| **mRMR** | 0.67 (15) | 0.97 (19) | 1.00 (435) | 0.82 (9) | 0.72 (9) |
| **Relief** | 0.52 (14) | 0.97 (97) | 0.87 (113) | **1.00 (176)** | 0.88 (248) |
| **RF** | 0.85 (205) | 0.57 (16) | 0.88 (206) | 0.87 (13) | 0.53 (16) |
| **IG** | 0.45 (9) | 0.67 (10) | 0.60 (10) | 0.73 (13) | 0.67 (10) |
| **Poll (ranking)** | 0.45 (21) | 0.77 (13) | 0.87 (252) | 0.67 (15) | 0.67 (17) |
| **Clinical** | 0.90 (33) | 0.77 (33) | 0.67 (33) | 0.70 (33) | 0.67 (33) |

Table D.11: AUC and number of features used for each one of the tested combinations of FS method and ML-based classifier during the 10-fold cross-validation for chronic pneumonitis prediction.

|  | **kNN** | **GLM** | **NB** | **SVM** | **ANN** |
| --- | --- | --- | --- | --- | --- |
| **CFS** | 0.59 (4) | 0.79 (4) | 0.72 (4) | 0.76 (4) | 0.79 (4) |
| **Chi-squared** | 0.52 (30) | 0.84 (30) | 0.81 (30) | 0.70 (30) | 0.82 (30) |
| **Boruta** | 0.55 (14) | 0.82 (14) | 0.76 (14) | 0.79 (14) | 0.77 (14) |
| **Poll (subsetting)** | 0.49 (11) | 0.81 (11) | 0.72 (11) | 0.79 (11) | 0.81 (11) |
| **mRMR** | 0.53 (9) | 0.86 (34) | 0.73 (101) | 0.70 (30) | **0.90 (32)** |
| **Relief** | 0.58 (403) | 0.64 (77) | 0.69 (222) | 0.59 (296) | 0.61 (199) |
| **RF** | 0.53 (5) | 0.83 (38) | 0.80 (156) | 0.83 (199) | 0.74 (173) |
| **IG** | 0.57 (5) | 0.79 (18) | 0.75 (20) | 0.72 (20) | 0.83 (18) |
| **Poll (ranking)** | 0.55 (12) | 0.84 (18) | 0.83 (121) | 0.85 (145) | 0.73 (99) |
| **Clinical** | 0.57 (33) | 0.57 (33) | 0.57 (33) | 0.56 (33) | 0.59 (33) |

Table D.12: AUC and number of features used for each one of the tested combinations of FS method and ML-based classifier during external validation for chronic pneumonitis prediction.

|  | **kNN** | **GLM** | **NB** | **SVM** | **ANN** |
| --- | --- | --- | --- | --- | --- |
| **CFS** | 0.62 (4) | 0.74 (4) | 0.47 (4) | 0.73 (4) | 0.74 (4) |
| **Chi-squared** | 0.70 (30) | 0.79 (30) | 0.68 (30) | 0.59 (30) | 0.71 (30) |
| **Boruta** | 0.63 (14) | 0.70 (14) | 0.50 (14) | **0.80 (14)** | 0.71 (14) |
| **Poll (subsetting)** | 0.77 (11) | 0.78 (11) | 0.47 (11) | 0.67 (11) | 0.79 (11) |
| **mRMR** | 0.52 (9) | 0.75 (34) | 0.74 (101) | 0.80 (30) | 0.73 (32) |
| **Relief** | 0.58 (403) | 0.58 (77) | 0.53 (222) | 0.53 (296) | 0.55 (199) |
| **RF** | 0.55 (5) | 0.63 (38) | 0.57 (156) | 0.57 (199) | 0.64 (173) |
| **IG** | 0.50 (5) | 0.76 (18) | 0.44 (20) | 0.68 (20) | 0.79 (18) |
| **Poll (ranking)** | 0.34 (12) | 0.76 (18) | 0.46 (121) | 0.68 (145) | 0.51 (99) |
| **Clinical** | 0.48 (33) | 0.67 (33) | 0.65 (33) | 0.58 (33) | 0.63 (33) |

1. P. N. Bennett, “Assessing the calibration of naive bayes posterior estimates,” Carnegie-Mellon Univ Pittsburgh PA School of Computer Science, 2000. [↑](#footnote-ref-1)
2. Y. Huang, W. Li, F. Macheret, R. A. Gabriel, and L. Ohno-Machado, “A tutorial on calibration measurements and calibration models for clinical prediction models,” J. Am. Med. Inform. Assoc., vol. 27, no. 4, pp. 621–633, 2020. [↑](#footnote-ref-2)
3. [G. C. M. Siontis, I. Tzoulaki, P. J. Castaldi, and J. P. A. Ioannidis, “External validation of new risk prediction models is infrequent and reveals worse prognostic discrimination,” *J. Clin. Epidemiol.*, vol. 68, no. 1, pp. 25–34, Jan. 2015, doi: 10.1016/j.jclinepi.2014.09.007.](https://www.zotero.org/google-docs/?iP8xZL) [↑](#footnote-ref-3)
4. H. A. Abu Alfeilat et al., “Effects of Distance Measure Choice on K-Nearest Neighbor Classifier Performance: A Review,” Big Data, vol. 7, no. 4, pp. 221–248, Dec. 2019, doi: 10.1089/big.2018.0175. [↑](#footnote-ref-4)
